# Geographic Variation in Plant Community Structure of Salt Marshes: Species, Functional and Phylogenetic Perspectives

**DOI:** 10.1371/journal.pone.0127781

**Published:** 2015-05-26

**Authors:** Hongyu Guo, Scott A. Chamberlain, Eran Elhaik, Inder Jalli, Alana-Rose Lynes, Laurie Marczak, Niv Sabath, Amy Vargas, Kazimierz Więski, Emily M. Zelig, Steven C. Pennings

**Affiliations:** 1 Department of Biology and Biochemistry, University of Houston, Houston, Texas, United States of America; 2 Department of Ecology and Evolutionary Biology, Rice University, Houston, Texas, United States of America; Fudan University, CHINA

## Abstract

In general, community similarity is thought to decay with distance; however, this view may be complicated by the relative roles of different ecological processes at different geographical scales, and by the compositional perspective (e.g. species, functional group and phylogenetic lineage) used. Coastal salt marshes are widely distributed worldwide, but no studies have explicitly examined variation in salt marsh plant community composition across geographical scales, and from species, functional and phylogenetic perspectives. Based on studies in other ecosystems, we hypothesized that, in coastal salt marshes, community turnover would be more rapid at local versus larger geographical scales; and that community turnover patterns would diverge among compositional perspectives, with a greater distance decay at the species level than at the functional or phylogenetic levels. We tested these hypotheses in salt marshes of two regions: The southern Atlantic and Gulf Coasts of the United States. We examined the characteristics of plant community composition at each salt marsh site, how community similarity decayed with distance within individual salt marshes versus among sites in each region, and how community similarity differed among regions, using species, functional and phylogenetic perspectives. We found that results from the three compositional perspectives generally showed similar patterns: there was strong variation in community composition within individual salt marsh sites across elevation; in contrast, community similarity decayed with distance four to five orders of magnitude more slowly across sites within each region. Overall, community dissimilarity of salt marshes was lowest on the southern Atlantic Coast, intermediate on the Gulf Coast, and highest between the two regions. Our results indicated that local gradients are relatively more important than regional processes in structuring coastal salt marsh communities. Our results also suggested that in ecosystems with low species diversity, functional and phylogenetic approaches may not provide additional insight over a species-based approach.

## Introduction

Understanding variation in community structure across geographical scales is one of the major goals in ecology and biogeography. Community similarity decreases as geographical distance increases, a pattern that has been recognized in ecology and biogeography for several decades [1,2], and that provides an important measure of community compositional variation in ecosystems [2,3,4]. The decrease of similarity between two ecological communities with distance may be due to niche-based processes such as environmental selection and local adaptation [5], spatial configuration [6] or organisms’ limited dispersal abilities [7], and these processes may not be mutually exclusive in natural ecological communities [8,9]. The relationship between geographical distance and community similarity could be influenced by different ecological processes at different geographical scales [8,10,11], thus examining the variation patterns of community structure across geographical scales is essential for better understanding the relative importance of different ecological factors in shaping communities.

Community distribution patterns are traditionally studied with a species-based approach, but recently there has been increasing interest in examining community structure and diversity from functional and phylogenetic perspectives [12,13,14]. As individuals in ecological communities interact with the environment and other individuals mainly through their functional traits which determine when and where these individuals can exist [15,16], studies based on functional traits could improve our mechanistic understanding of community assembly [14,17]. Moreover, there has also been increasing attention paid to evaluating evolutionary influences on community structure by incorporating phylogenetic information into community ecology [12,13]. Studies with combined approaches from species, functional and phylogenetic perspectives have enabled ecologists to obtain further insights into the processes shaping community structure [11,18,19].

Salt marshes are widely distributed in coastal areas worldwide, outside the tropics which are dominated by mangroves, occurring wherever wave action is modest enough to permit the accumulation of sediments and rooted plants [20]. In particular, they are the dominant intertidal habitat along the Atlantic and Gulf Coasts of the United States [21]. Salt marshes contain a relatively simple community of plant species that is strongly influenced by abiotic factors, such as flooding and soil porewater salinity, that vary across the marsh platform [22,23,24]. These abiotic factors, however, also vary geographically. In particular, salt marshes experience different tidal regimes between the southern Atlantic and Gulf Coasts in the United States. The southern Atlantic Coast has a regular semi-diurnal flooding schedule, with tidal amplitudes of 2–3 m [25]. In contrast, the Gulf Coast experiences diurnal tides of only ~0.5 m in amplitude [26], and this micro-tide regime is often overshadowed by meteorological and geomorphologic conditions, resulting in a relatively irregular tidal regime [27,28]. The different tidal regimes between the southern Atlantic and Gulf Coasts may favor different ecological processes, producing different diversity patterns in salt marsh plant communities [29].

Although salt marshes have been studied extensively worldwide, we are aware of no studies that have explicitly examined variation in salt marsh plant community composition across geographical scales, and from multiple compositional perspectives (e.g. species, functional groups and phylogenetic lineages). We hypothesized that community turnover would be more rapid at the local scale than at larger geographical scales, as the strong abiotic gradients from high to low intertidal elevations would play relatively important roles in structuring communities at the local scale. We further hypothesized that community turnover patterns would diverge among compositional perspectives, with a greater distance decay at the species level than at the functional or phylogenetic levels. We tested our hypotheses by investigating geographic variation in plant community structure of salt marshes on the southern Atlantic and Gulf Coasts in the United States, from species, functional and phylogenetic perspectives. We classified salt marsh plant species in three different ways: as individual species, as functional groups, and as taxonomic groups based on phylogeny. Specifically, we examined the characteristics of plant community composition at each salt marsh site, how plant community similarity decayed with distance within individual salt marshes (along transects across elevation) versus among sites in Georgia and Texas (representing the southern Atlantic and western Gulf Coasts, respectively), and how plant community similarity differed among sites at the regional scale.

## Material and Methods

### Study sites and datasets

We analyzed datasets of salt marsh plant community composition from 59 sites in Georgia (GA) and 49 sites in Texas (TX) (Kunza & Pennings 2008; data are available from the GCE-LTER website (http://gce-lter.marsci.uga.edu/) as datasets PLT-GCET-0802a and PLT-GCET-0711). Data were collected in April and May 2005 for TX sites, and June and July 2005 for GA sites.

Within each region (GA or TX), sites were chosen to include a range of mainland, barrier island, and back-barrier island (hammock) locations. All sites were dominated by salt marsh vegetation. At each site, plant community composition was surveyed along a transect that began at the lower elevational limit of vegetation and continued perpendicularly to the water's edge up to the lower edge of the shrub community at the upper marsh border (defined by the presence of a non-stunted shrub). The transects in TX had average length of 85 ± 6 m; the transects in GA had average length of 122 ± 14 m.

Along each transect, 0.5 × 0.5 m quadrats were located at 1 m intervals, and the percent cover of each plant species within each quadrat was scored on a six point scale: absent, present but less than 5%, between 5% and 12%, between 13% and 25%, between 26% and 50%, and greater than 50% [29]. Scores were later converted to percent cover values using the midpoint of each cover range. Plant species were identified following USDA Plants website (www.plants.usda.gov).

### Plant functional groups

We collected data on 13 plant traits from the USDA Plants website (www.plants.usda.gov) and local floras. Three traits were numerical (maximum height, seed mass, and USDA regional wetland indicator status), Ten traits were in nominal categories (growth form [30], longevity (annual, biennial or perennial), C3 versus C4 photosynthetic pathway, monocot versus dicot, tall versus short, and clonal, herbaceous, succulent, parasitic, and scramble or not). These traits have commonly been used to define functional groups, and trait data were available for all species in this study.

We used the list of plant traits to calculate the trait dissimilarity between each pair of species. Let T^k^_i_ be the value of trait k in species i. Let d^k^_i,j_ be the trait dissimilarity between species i and j in trait k. For traits in nominal categories, d^k^_i,j_ = 0 if T^k^_i_ is equal to T^k^_j_, and 1 if T^k^_i_ is not equal to T^k^_j_. For numerical traits, let d^k^_i,j_ = absolute (T^k^_i_—T^k^_j_) / (highest T^k^—lowest T^k^). Let D_i, j_ = average (d^k^_i, j_) be the mean dissimilarity between species i and j. We used the UPGMA (Unweighted Pair Group Method with Arithmetic mean) clustering method to construct a dendrogram of species relationship based on functional traits. Results of the clustering analysis were robust against the exclusion of particular traits.

Based on the dendrogram, we identified ten functional groups. We refer to each using a shorthand that identifies one or two major features that distinguish each group: 1) parasitic vines, 2) perennial succulents, 3) annual succulents, 4) annual dicots, 5) rushes + sedges, 6) tall grasses, 7) short grasses, 8) non-parasitic vines, 9) perennial dicots, and 10) shrubs (S1 Table). The number of species in each functional group ranged from one to five.

### Plant taxonomic groups based on phylogeny

Using the online interface Phylomatic [31] and the angiosperm supertree by Davies *et al*. [32], we constructed a phylogeny including all species. Since phylogenies constructed using the “maximally resolved seed plant tree” and the “conservative seed plant tree” yielded similar results as the supertree by Davies *et al*. [32], we decided to just use the latter. We identified ten taxonomic groups according to the clusters in the phylogeny: 1) Lamiales [33], 2) Solanales, 3) Asclepidaceae + Gentianaceae, 4) Asteraceae + Apiaceae, 5) Bataceae, 6) Plumbaginaceae, 7) Cactaceae + Aizoaceae, 8) Amaranthaceae [33], 9) Poaceae, 10) Cyperaceae + Juncaceae (S1 Table). The number of species in each taxonomic group ranged from one to nine.

### Community compositional variation in salt marshes

We first studied the characteristics of community compositional variation in salt marshes from species, functional and phylogenetic perspectives, by analyzing plant composition patterns along transects in the salt marshes of GA and TX with the three classification approaches. For each salt marsh site, we normalized the data of species coverage along each transect by dividing the transect into 22 intervals of equal length spanning the entire length of the transect, and binning the plots into these intervals. Species coverages were averaged within each bin, and then averaged for each bin at the same position on the transects across all the sites within each region. To normalize the coverage data of functional group and taxonomic group, we averaged the coverage data of functional or taxonomic groups within each bin, and then averaged the coverage data within each region as above.

### Distance-decay of community similarity

We examined variation in the patterns of distance-decay of community similarity by analyzing community compositional data (percent cover) for species, functional groups, and taxonomic groups at different geographical scales. We used community dissimilarity (1- similarity), since it also directly represents community β-diversity, which indicates the characteristic of community structure variation. All analyses were performed using R [34].

To examine the distance-decay of community similarity within sites, we analyzed the relationship of the physical (Euclidean) distance of all pairs of plots within a site versus their community dissimilarity (Bray-Curtis dissimilarity). For each site, we calculated the slope of the linear regression between physical distance and plot dissimilarity, and we estimated the standard error and range of the slopes for GA and TX respectively. We compared the slopes among the three classification approaches for GA and TX respectively, using non-parametric Kruskal-Wallis tests, due to lack of normality of residuals. For each site, we also performed Mantel tests with the Pearson method to calculate the correlation coefficient (Mantel r) between physical distance and plot dissimilarity, using the vegan package in R [35]. Significance of each Mantel test was evaluated by randomizing the dataset 10,000 times, then comparing the observed Mantel r value against the distribution of Mantel r values obtained from the randomized datasets. We compared the Mantel r values among the three classification approaches using ANOVA for GA and TX respectively. We performed post-hoc Tukey HSD tests to compare means following a significant ANOVA result.

To examine the distance-decay of community similarity among sites within each region (GA or TX), we analyzed the relationship of the physical distance between the sites (calculated from GPS coordinates using Euclidean distance) versus the community dissimilarity (Bray-Curtis dissimilarity) of all pairs of the sites (calculated based on the mean plant composition of all plots within each site). For each region (GA or TX), we calculated the slope of the linear regression between physical distance and site dissimilarity [4,11]. For each region (GA or TX), we also performed Mantel tests with the Pearson method to calculate the correlation coefficient (Mantel r) between physical distance and site dissimilarity [11] as described above. Significance of the Mantel tests was estimated using randomization tests (10,000 times) as described above.

Finally, we examined the patterns of overall community dissimilarity (β-diversity) of salt marshes in GA and TX by comparing the Bray-Curtis dissimilarity of all pairs of sites within GA, all pairs of sites within TX, and all between-region pairs of sites (GA versus TX), for species, functional groups and taxonomic groups respectively. We used ANOVAs followed by post-hoc Tukey HSD tests to compare means.

## Results

### Community compositional variation in salt marshes

**Fig 1:**
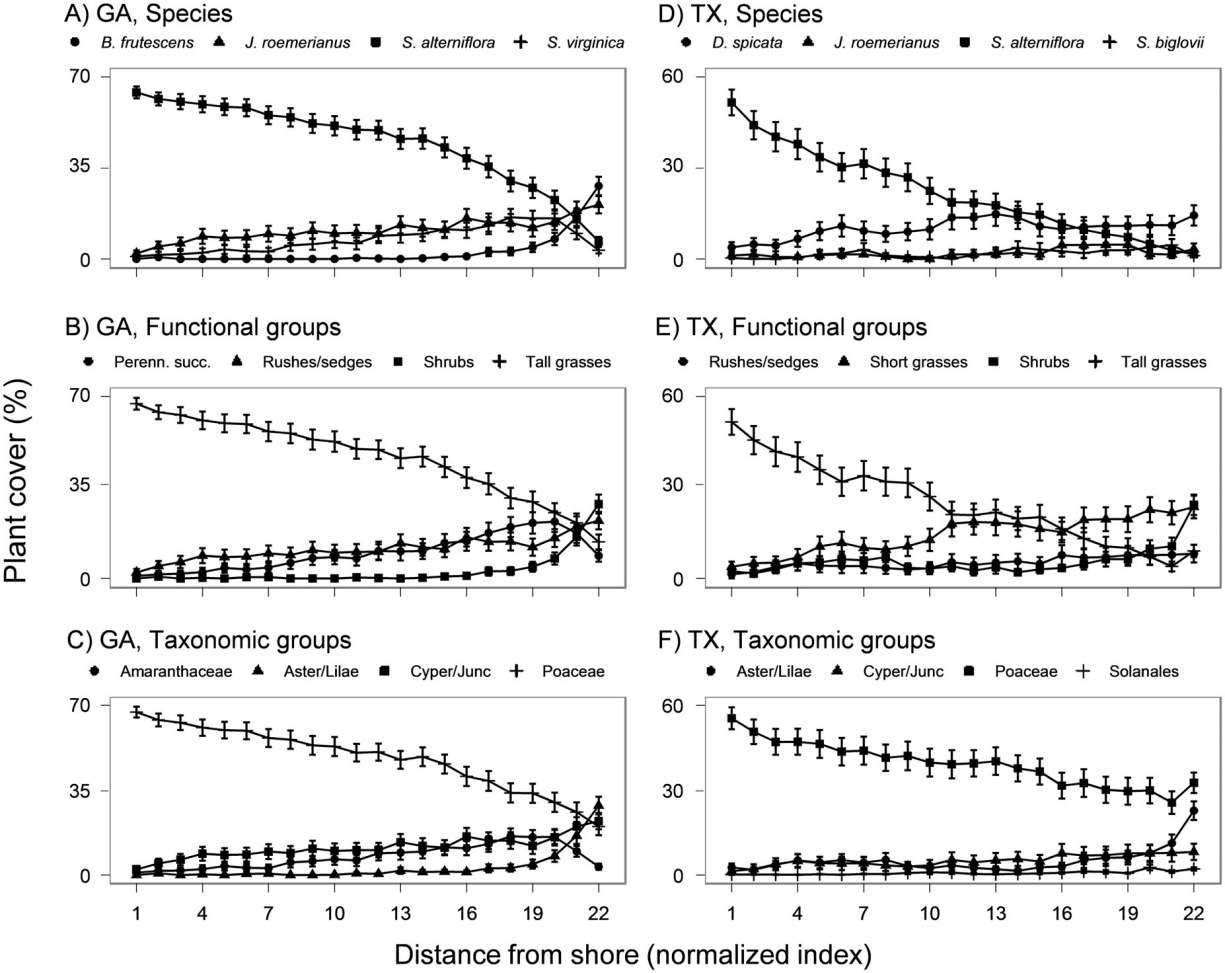
Community compositional variation in salt marshes presented by the four most dominant plant species, functional groups, and taxonomic groups respectively in GA and TX regions. Distance from shore was normalized to 22 bins for each transect, and mean plant cover was calculated within each normalized bin for each species, functional, or taxonomic group.

Patterns of plant community distribution in salt marshes tended to differ between GA and TX (Fig 1). In GA, most salt marshes were dominated by monospecific stands of the tall grass, *Spartina alterniflora*, which mostly occupied the shoreward end of the transects; at some sites, *S*. *alterniflora* covered over 90% of the transects (Fig 1A). Further inland, a transition zone began consisting of a mixture of *S*. *alterniflora* and perennial succulents such as *Batis maritima* and *Salicornia virginica*. At some sites, these succulents formed distinct monospecific stands that covered over 30% of the transects; at other sites, they were mixed with *Distichlis spicata*. The succulent zone was often bordered inland by a dense stand of the rush *Juncus roemerianus*, sometimes accompanied at the most inland portion of the transect by the shrub *Borrichia frutescens*. When vegetation percent cover data were plotted for functional (Fig 1B) or taxonomic (Fig 1C) groups, the resulting patterns were similar to the pattern for species.

In TX, salt marshes appeared to be less structured (Fig 1D, 1E and 1F). Although *S*. *alterniflora* again created monospecific stands on the shoreward end of the transects, it did not extend as far inland as in the salt marshes of GA (Fig 1D). A mixed zone of short grasses, such as *D*. *spicata* and *Monanthochloe littoralis*, and succulents, such as *B*. *maritima*, *S*. *virginica* and *Salicornia bigelovii*, occupied a wide range of the mid-marsh. *J*. *roemerianus* was present in TX, but occurred less frequently than in GA, and occurred in mixed species patches rather than large monospecific stands. A shrub zone was again present at the most inland portion of transects. Plotting vegetation percent cover data for functional groups emphasized the importance of short grasses in the salt marshes of TX (Fig 1E), and plotting vegetation percent cover data for taxonomic groups emphasized the dominance of grasses across the entire marsh landscape in the salt marshes of TX (Fig 1F).

### Distance-decay of community similarity within sites

**Fig 2:**
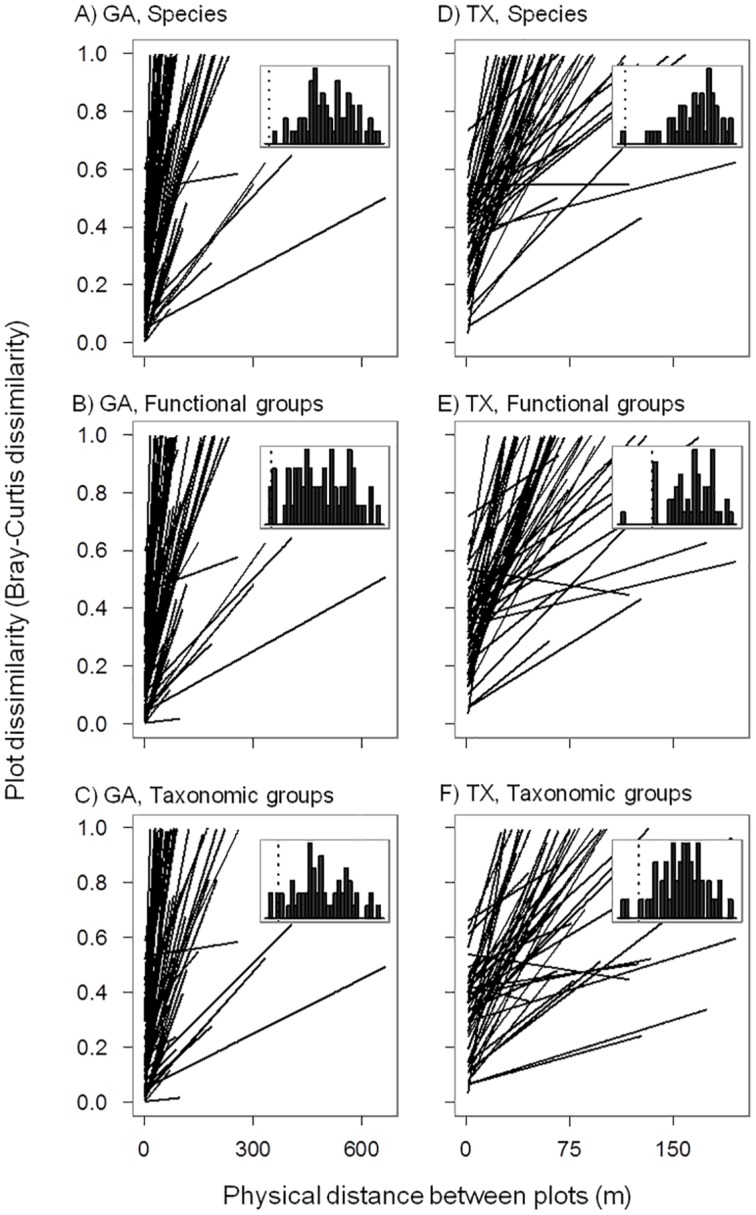
Relationship between plot distance and plot dissimilarity (Bray-Curtis dissimilarity) for each of the 59 transects in GA and the 49 transects in TX, based on species (Panel A and D), functional groups (Panel B and E), and taxonomic groups (Panel C and F) respectively. In each panel, the lines represent linear regression fit to the data for each transect (detailed data for each transect are not shown). In each panel, the frequency histrogram of Mantel r values (ranges from 0 to 1) from Mantel tests for each transect is shown (significant Mantel r values evaluated by randomization tests are to the right of the dashed line).

For most transects in both GA and TX, linear regression and Mantel tests showed that there tended to be positive relationship between plot distance and plot dissimilarity, regardless of the classification approaches (Table 1, Fig 2). In both GA and TX, the slopes of linear regression between plot distance and plot dissimilarity did not significantly differ among the three classification approaches (Table 1; GA: Kruskal-Wallis test *χ* ^2^ = 1.55, *P* = 0.46; TX: Kruskal-Wallis test *χ* ^2^ = 5.54, *P* = 0.06). The correlation coefficients from Mantel tests (Mantel r values) between plot distance and plot dissimilarity were not significantly different among the three classification approaches for sites in GA (ANOVA of Mantel r values: *F* = 0.56, *P* = 0.57). There was a difference in Mantel r values between plot distance and plot dissimilarity among the three classification approaches for sites in TX (ANOVA of Mantel r values: *F* = 5.43, *P*< 0.01), with higher Mantel r value for species than for taxonomic groups (Tukey HSD test, *P*< 0.05).

**Table 1:** Summary of relationships (slopes (β) from linear regression and Mantel r values) between physical distance and community dissimilarity within and among sites in GA and TX regions.

Region and scale	Classification approaches		
	Species	Functional groups	Taxonomic groups
GA			
Within sites (22–666 plots)	β: 0.010 (0.001) [0.0002, 0.045]	β: 0.009 (0.001) [0.0001, 0.045]	β: 0.008 (0.001) [0.0001, 0.045]
	r: 0.41 (0.02) [0.03, 0.76]	r: 0.40 (0.02) [0.07, 0.76]	r: 0.38 (0.02) [0.03, 0.76]
Among sites (59 sites)	β: 0.0000016	β: 0.0000017	β: 0.0000019
	r: 0.11	r: 0.12	r: 0.13*
TX			
Within sites (26–197 plots)	β: 0.011 (0.001) [-0.00001, 0.036]	β: 0.010 (0.001) [-0.0008, 0.036]	β: 0.008 (0.001) [-0.001, 0.036]
	r: 0.48 (0.02) [-0.001, 0.73]	r: 0.44 (0.02) [-0.07, 0.73]	r: 0.37 (0.02).[-0.07, 0.73]
Among sites (49 sites)	β: 0.00000029	β: 0.00000028	β: 0.00000018
	r: 0.15*	r: 0.14*	r: 0.10*

For within-site relationships, data are shown as mean (+se) [range]. For among-site relationships, asterisks indicate Mantel r values that were significantly different from zero (Randomization tests: *P* < 0.05).

### Distance-decay of community similarity among sites

For both GA and TX, the slopes of linear regressions between site distance and site dissimilarity were all positive, regardless of the classification approaches (Table 1). In GA, the Mantel r values between site distance and site dissimilarity were marginally significant for species and functional groups (Randomization tests: *P* = 0.08 and 0.06, respectively), and significant for taxonomic groups (Randomization tests: *P* = 0.04). In TX, the Mantel r values between site distance and site dissimilarity were all significant regardless of the classification approaches (Table 1).

Within each region (GA or TX), community composition tended to vary with much lower rates between sites than within sites, regardless of the classification approaches (Table 1). The slopes from the linear regressions between plot distance and plot dissimilarity within sites (0.8×10^–2^ to 1.1 ×10^–2^) were generally four to five orders of magnitude greater than the slopes from the linear regressions between site distance and site dissimilarity (1.8 × 10^–7^ to 1.9 × 10^–6^). The Mantel r values between plot distance and plot dissimilarity within each site (0.37 to 0.48) were around three-fold higher than the Mantel r values between site distance and site dissimilarity (0.10 to 0.15).

### Community dissimilarity (β-diversity) of salt marshes in GA and TX

**Fig 3:**
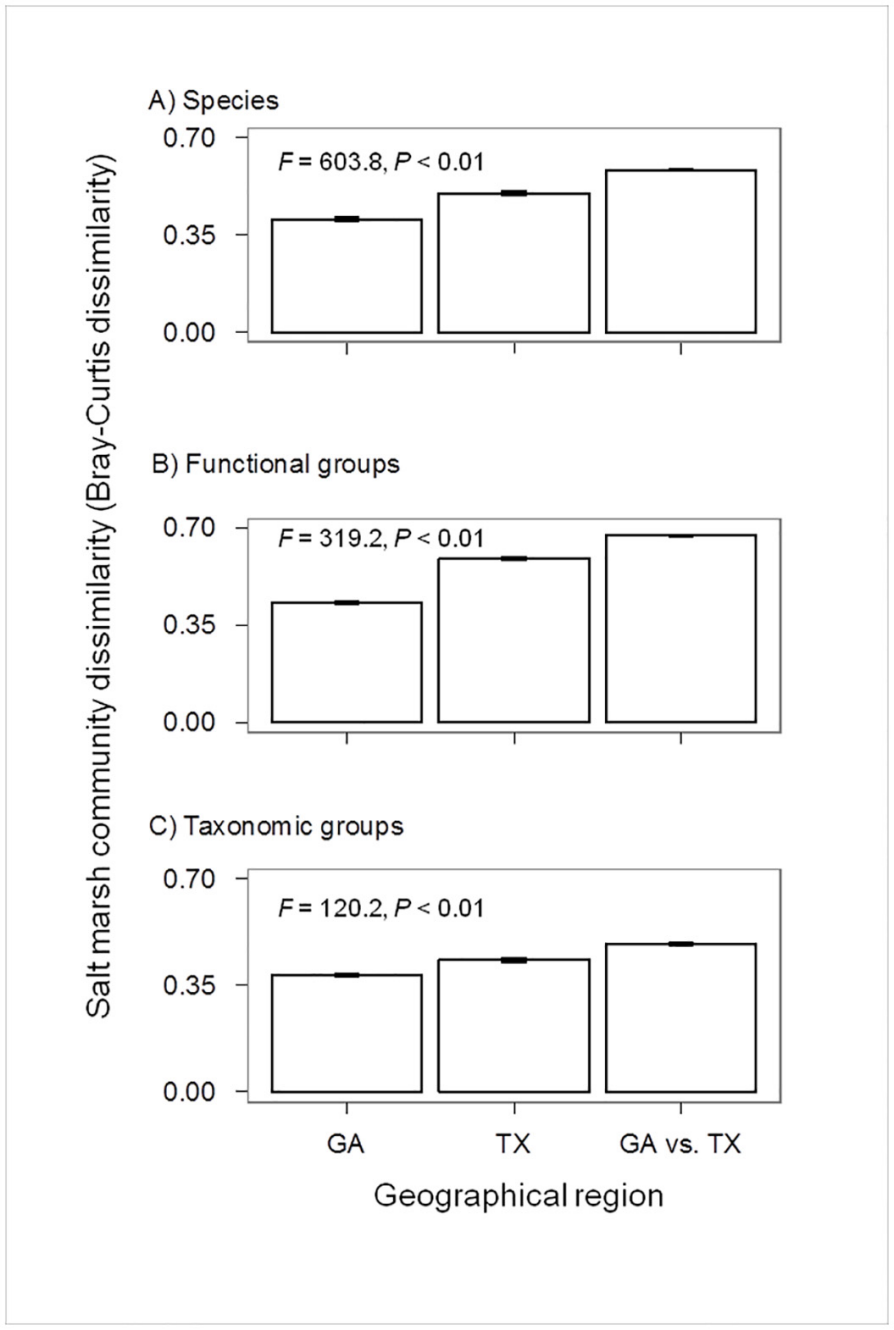
Salt marsh community dissimilarity (Bray-Curtis dissimilarity) of all pairs of sites in GA (Panel A), all pairs of sites in TX (Panel B), and all between-region pairs of sites (GA versus TX; Panel C). ANOVA results are shown for each classification approach. Salt marsh community dissimilarity was lowest in GA, intermediate in TX, and highest for the pairs of sites between GA and TX for all the classification approaches (Tukey HSD tests, *P* < 0.05).

Community dissimilarity (β-diversity) of salt marshes differed in the comparisons for all pairs of sites within GA, all pairs of sites within TX, and all between-region pairs of sites (GA versus TX), regardless of the classification approaches (Fig 3; Species: ANOVA *F* = 603.8, *P* < 0.01; Functional groups: ANOVA *F* = 319.2, *P* < 0.01; Taxonomic groups: ANOVA *F* = 120.2, *P* < 0.01). For all the three classification approaches, community dissimilarity of salt marshes was lowest in GA, intermediate in TX, and highest for the pairs of sites between GA and TX (Tukey HSD tests, *P* < 0.05).

## Discussion

Our results supported our hypothesis that community turnover in coastal salt marshes would be more rapid at local versus larger geographical scales. Our results showed that there was strong variation in plant community composition within individual salt marsh sites across elevation regardless of the classification approaches, which is consistent with the previous studies on the distribution patterns of plant communities in salt marshes [21,23,36]. Salt marshes typically contain strong environmental gradients across elevation, such as flooding and soil porewater salinity gradients [21,37,38]. These environmental factors may interplay with biotic interactions between plant species (e.g. competition and facilitation), thereby mediating plant composition patterns in salt marshes [22,23,39], and resulting in strong variation in plant composition across intertidal elevation within salt marsh sites.

In contrast, our results indicated that community similarity decayed with distance much slower across salt marsh sites within each region (GA or TX), regardless of the classification approaches. In other words, plant community composition of salt marshes among different sites within GA or TX tended to be relatively similar. This reflects the fact that coastal salt marshes are relatively simple communities comprised of species that have wide geographical distributions [25,28,29,40]. Results from this study and previous investigations suggested that coastal salt marshes in GA or TX are dominated by a relatively small groups of salt marsh plant species such as *S*. *alterniflora*, *J*. *roemerianus*, *B*. *maritima*, *S*. *virginica* and *D*. *spicata* [25,28,29,41], which may lead to relatively weak turnover in community composition among salt marsh sites within either GA or TX.

Even the between-region (GA versus TX) dissimilarity was only slightly higher than the between-site dissimilarity within each region (GA or TX), which suggests considerable similarity of the dominant vegetation in salt marshes between GA and TX [25,28,29]. The variation in community dissimilarity that appeared within each region and between the regions might be due to the differences in abiotic conditions across spatial scales (not measured in this study) or to stochastic processes associated with dispersal and colonization of the salt marsh species.

On the other hand, our results revealed that the overall community dissimilarity (β-diversity) of salt marshes was lower in GA than in TX. This pattern indicated that salt marsh plant communities tended to be more diverse across TX than in GA, which is consistent with observations in previous studies of plant community composition in the two regions [25,29,42]. The higher community dissimilarity of the salt marshes across TX might be due to the relatively irregular micro-tide regime in the western Gulf Coast. In TX, the more variable tidal regime tends to produce heterogeneous environmental conditions, because extended periods of flooding or exposure can produce extremely waterlogged and extremely dry, hypersaline soils, respectively [28,29]. Higher environmental heterogeneity would be expected to promote more diverse community structure [43,44,45]. Thus, the higher community dissimilarity of the salt marshes in TX versus GA likely indicated the important influence of the different tidal regimes (micro-tidal in TX versus macro-tidal in GA) on salt marsh community structure in the two geographical regions, coupled with other abiotic differences between the regions.

The results from the three species classification methods did not support our hypothesis that community turnover patterns would diverge among compositional perspectives. Instead, our results indicated that the three species classification methods generally showed similar patterns, suggesting that in low-diversity systems, such as salt marshes, using functional groups based on traits or taxonomic groups based on phylogeny may not substantially enhance our understanding of the plant community structure patterns. Although functional and phylogeny-based approaches have been applied successfully in species-rich systems, such as tropical forests [46,47,48], some studies in systems with relatively small number of species have suggested that a functional group approach might not be more helpful in interpreting vegetation patterns than a species-level approach. For example, Sullivan and Zedler [49] found that each plant species (8 species) in a salt marsh tended to function differently, and they suggested that uncritically assigning tidal marsh halophytes into functional groups might confuse rather than clarify the interpretation of study results. Epstein *et al*. [50] found that aggregating arctic tundra species (15 species) into functional groups (7 types) appeared not to substantially alter the results of analyses of whole ecosystem properties. Pavoine *et al*. [51] showed that the phylogenetic diversity in a coastal marsh plain (56 species) might not provide better insights than species richness in understanding the ecological processes shaping the local plant community. Thus, more caution may be needed when applying functional and phylogenetic approaches to study ecosystems with relatively low species diversity, such as salt marshes.

Overall, our results showed that the turnover rates of plant community in coastal salt marshes varied across geographical scales. Ecological factors at local scales (such as abiotic gradients across intertidal elevation) were relatively more important than processes at regional scales (such as dispersal) in shaping plant community structure in coastal salt marshes. Moreover, greater community dissimilarity in TX than in GA suggested that similar communities may be structured differently in different geographical regions. Our results also showed that, contrary to our original hypothesis, community turnover patterns from species, functional and phylogenetic perspectives converged. In ecosystems such as salt marshes with low species diversity, functional and phylogenetic approaches may not provide additional insight over a species-based approach.

## Supporting Information

S1 Table List of species and corresponding functional and taxonomic (based on phylogeny) groups. Plant species were identified following USDA Plants website (www.plants.usda.gov).
(DOCX)
