# Supplementary material for: Geographic Variation in Plant Community Structure of Salt Marshes: Species, Functional and Phylogenetic Perspectives
Source: PLoS One. 2015 May 26;10(5):e0127781. doi: 10.1371/journal.pone.0127781 (PMC4444317; doi:10.1371/journal.pone.0127781)
Supplement: S1 Table — Plant species were identified following USDA Plants website (www.plants.usda.gov). (DOCX) [file pone.0127781.s001.docx]

**Supporting Information**

**S1 Table: List of species and corresponding functional and taxonomic groups**

S1 Table. List of species and corresponding functional and taxonomic (based on phylogeny) groups. Plant species were identified following USDA Plants website ([www.plants.usda.gov](http://www.plants.usda.gov)). How to name species that were historically in the genus *Salicornia* is currently under debate, with different authorities giving different names for the same species; we have retained the traditional names used in the literature.

| Species | Functional groups | Taxonomic groups |
| --- | --- | --- |
| *Agalinis maritima* | Annual dicots | Lamiales |
| *Aster spp.* | Perennial dicots | Asteraceae/ Apiaceae |
| *Symphyotrichum tenuifolium* | Perennial dicots | Asteraceae/ Apiaceae |
| *Avicennia germinans* | Shrubs | Lamiales |
| *Baccharis halimifolia* | Shrubs | Asteraceae/ Apiaceae |
| *Batis maritima* | Perennial succulents | Bataceae |
| *Borrichia frutescens* | Shrubs | Asteraceae/ Apiaceae |
| *Cuscuta indecora* | Parasitic vines | Solanales |
| *Cynanchum angustifolium* | Non-parasitic vines | Asclepidaceae/Gentianaceae |
| *Distichlis spicata* | Short grasses | Poaceae |
| *Eustoma exaltatum* | Annual dicots | Asclepidaceae/Gentianaceae |
| *Fimbristylis spp.* | Rushes + Sedges | Cyperaceae/Juncaceae |
| *Helianthus annuus* | Annual dicots | Asteraceae/ Apiaceae |
| *Iva frutescens* | Shrubs | Asteraceae/ Apiaceae |
| *Juncus roemerianus* | Rushes + Sedges | Cyperaceae/Juncaceae |
| *Juncus spp.* | Rushes + Sedges | Cyperaceae/Juncaceae |
| *Lilaeopsis sp.* | Perennial dicots | Asteraceae/ Apiaceae |
| *Limonium carolinianum* | Perennial dicots | Plumbaginaceae |
| *Lycium carolinianum* | Shrubs | Solanales |
| *Rayjacksonia phyllocephala* | Annual dicots | Asteraceae/ Apiaceae |
| *Monanthochloe littoralis* | Short grasses | Poaceae |
| *Opuntia humifusa* | Perennial succulents | Cactaceae/Aizoaceae |
| *Sabatia arenicola* | Annual dicots | Asclepidaceae/Gentianaceae |
| *Salicornia bigelovii* | Annual succulents | Amaranthaceae |
| *Salicornia virginica* | Perennial succulents | Amaranthaceae |
| *Schoenoplectus americanus* | Rushes + Sedges | Cyperaceae/Juncaceae |
| *Schoenoplectus robustus* | Rushes + Sedges | Cyperaceae/Juncaceae |
| *Sesuvium portulacastrum* | Perennial succulents | Cactaceae/Aizoaceae |
| *Solidago sempervirens* | Perennial dicots | Asteraceae/ Apiaceae |
| *Spartina alterniflora* | Tall grasses | Poaceae |
| *Spartina bakeri* | Tall grasses | Poaceae |
| *Spartina cynosuroides* | Tall grasses | Poaceae |
| *Spartina patens* | Tall grasses | Poaceae |
| *Spartina spartinae* | Tall grasses | Poaceae |
| *Sporobolus virginicus* | Short grasses | Poaceae |
| *Suaeda linearis* | Annual succulents | Amaranthaceae |

**References**

Davies TJ, Barraclough TG, Chase MW, Soltis PS, Soltis DE, et al. (2004) Darwin's abominable mystery: Insights from a supertree of the angiosperms. Proceedings of the National Academy of Sciences of the United States of America 101: 1904-1909.

Webb CO, Donoghue MJ (2005) Phylomatic: tree assembly for applied phylogenetics. Molecular Ecology Notes 5: 181-183.

The Angiosperm Phylogeny Group (2009) An update of the Angiosperm Phylogeny Group classification for the orders and families of flowering plants: APG III. Botanical Journal of the Linnean Society 161: 105-121.
